# Synergetic Effects of Ferrite Content and Tempering Temperature on Mechanical Properties of a 960 MPa Grade HSLA Steel

**DOI:** 10.3390/ma11102049

**Published:** 2018-10-20

**Authors:** Shaoyang Wang, Hao Yu, Tao Zhou, Lu Wang

**Affiliations:** 1 School of Materials Science and Engineering, University of Science and Technology Beijing, No. 30 Xueyuan Road, Haidian District, Beijing 100083, China; b20140539@xs.ustb.edu.cn (S.W.); taozhou1111@gmail.com (T.Z.); 2 National Center for Materials Service Safety, University of Science and Technology Beijing, No. 30 Xueyuan Road, Haidian District, Beijing 100083, China; wangluyoujia@gmail.com

**Keywords:** HSLA steel, ferrite content, tempering temperature, tensile, strain hardening, impact

## Abstract

The synergetic effects of ferrite content and tempering temperature on the mechanical properties of a Q960E steel have been investigated in detail to obtain the optimal combination of strength, ductility, and toughness for ultrahigh strength steels. After quenching from different temperatures between 790 to 900 °C, the ferrite content in the microstructure containing martensite varies from 56 vol% to 0, and then the specimens were tempered at 180 °C and 450 °C, respectively. High ferrite content reduces both yield and tensile strengths based on the law of mixtures. The tensile strength decreases with the increase of tempering temperature, while the change of yield strength is affected by the ferrite content. When tempering at low temperature, specimens with various ferrite content show different strain hardening behaviors, and the ferrite improves the elongation but deteriorates the toughness with different fracture mechanisms due to the strength difference between ferrite and martensite. Tempering at high temperature increases the ferrite–martensite co-deformation, resulting in the same strain hardening behavior for all specimens and the ferrite is benefit for both elongation and impact properties with similar fracture mechanisms. Moreover, the single martensite with homogeneous microstructure is essential for better toughness.

## 1. Introduction

High-strength low-alloy (HSLA) steels are widely used for construction machinery, buildings, pressure vessels, and many other fields due to their high strength, good low temperature toughness, excellent ductility, and weldability. However, with the development of ultrahigh strength steels, toughness and deformability usually deteriorate, so it is essential to balance the mechanical properties by precise metallurgical design consisting of chemical composition and microstructural constituents [1,2]. Generally, a dual-phase (DP) microstructure containing ferrite and martensite is known as one of the optimal microstructures to improve the strength with good ductility. In these steels the soft phase ferrite holds good deformability as well as cleavage fracture resistance, meanwhile, the volume fraction of the hard phase martensite determines the strength level [3,4]. Nevertheless, for the ultrahigh strength regime beyond 800 MPa, damage mechanisms of these steels shift and uniform elongation decreases with the increasing volume fraction of hard phase martensite [5], meantime the toughness is impacted by the high fraction of martensite [6]. Many strategies have been adopted to improve the ductility and impact properties of these ultrahigh strength DP steels, such as refining microstructure [7], alloying [8], design of new processing methods [9], etc.

After intercritical quenching, tempering is usually applied to modify the mechanical properties of DP steels [10]. The effects of tempering on the microstructure and mechanical properties of DP steels have been reported, and reveal that the tempering characteristics of DP steels are affected by chemical composition, volume fraction of martensite, retained austenite, and tempering parameters [11]. Yield behaviors are significantly affected by tempering temperatures, where DP steels perform discontinuous yielding when tempering at over 300 °C and the yield strength increases first and then decreases with the increase of tempering temperature, while tensile strength and elongation change slightly until tempering at high temperatures [12,13,14]. Li et al. [15] found that tensile strength had a linear relationship with the nanohardness ratio of martensite and ferrite. In addition, the reduction of the nanohardness difference between the two phases could effectively retard the void formation in martensite [16]. However, most of the work to date has been focused on the influence of tempering parameters on DP steels with a certain amount of constituent phases, and there is limited study addressing the synergetic effects of ferrite content and tempering temperature on the mechanical properties of DP steels.

In the present study, a Q960E HSLA steel with a hot-rolled microstructure of ferrite and pearlite was quenched from different intercritical temperature to acquire various ferrite/martenstie ratios in the microstructure and then tempered at 180 °C and 450 °C, respectively. The microstructure evolution are characterized and the effects of ferrite content on tensile and impact behaviors under different tempering conditions are discussed, and finally the fracture mechanisms of tensile and impact processes are analyzed in detail. This work provides valuable guidance in improving heat treatment to obtain the optimal combination of strength, ductility, and toughness in HSLA steels.

## 2. Experimental Procedure

A HSLA steel supplied by Ma Steel (Anhui, China) was selected in this study with the chemical composition of Q960E steel as shown in Table 1. The as-received steel is a 12 mm-thickness hot-rolled plate with a microstructure of ferrite and perlite; the critical temperatures A_c1_ and A_c3_ of the plate, measured by thermal dilatometry, are 730 °C and 860 °C, respectively. Specimens, 70 mm × 60 mm × 12 mm in size, wire-electrode cut from the plate, were subjected to quenching and tempering heat treatment schedules in a box-type resistance furnace. Specifically, in order to obtain different amounts of ferrite, the specimens were held at temperature of 790 °C, 810 °C, 830 °C, 850 °C, and 900 °C for 1 h, respectively, and then quenched in cold water to obtain martensite transformation. Thereafter, the specimens were tempered at 180 °C and 450 °C for 1 h, respectively.

For metallographic analysis, the specimens were prepared by wire cut electrical discharge machining with the observed surface parallel to the rolling plane, and then mechanically grinded, polished, and etched with 4% nital. The microstructure of the specimens after heat treatment was analyzed by light microscope (Carl Zeiss Microscopy GmbH, Jena, Germany) and SEM (Zeiss Ltd., Braunschweig, Germany). The volume fraction of ferrite was determined by the Image Pro-Plus 6.0 software (Media Cybernetics, Inc, Washington, WA, USA) based on the optical microscope images [17]. For further microstructural analysis, TEM analysis was also carried out on thin foils. These specimens were prepared by cutting thin wafers of 0.4 mm thickness and grinding them to ~60 μm. Discs of 3 mm diameter were punched from the wafers and electropolished with a solution of 4% perchloric acid in alcohol at −20 °C kept by liquid nitrogen. These foils were examined by a JEM-2100 TEM (JEOL Ltd., Tokyo, Japan) operated at 200 kV.

The mechanical properties of the specimens after heat treatment were assessed by tensile and Charpy V-notched impact tests. For tensile tests (Shanghai Xieqiang Instrument Technology Co., Ltd., Shanghai, China), specimens with round cross-section of 5 mm in diameter and 25 mm of original gauge length parallel to the rolling direction were machined according to Chinese standard GB/T 228.1-2010 [18]. Tensile tests were conducted at ambient temperature on a universal tensile testing machine of 200 kN capacity at a crosshead speed of 1 mm/min. According to Chinese standard GB/T 229-2007 [19], impact tests (Shanghai Xieqiang Instrument Technology Co., Ltd., Shanghai, China) were carried out at −40 °C on standard Charpy V-notch bars with dimensions of 10 mm × 10 mm × 55 mm and the length vertical to the rolling direction using a standard pendulum-type impact testing machine. Both of the tensile and impact tests were carried out on three specimens, and after that, the fracture surfaces were analyzed under SEM. In order to study the crack propagation characteristics, the fracture surfaces, after being analyzed, were electroplated with nickel, subsequently cut vertically to the V-notch, and finally prepared for SEM analysis.

## 3. Results and Discussion

### 3.1. Microstructural Investigation

**Figure 1:**
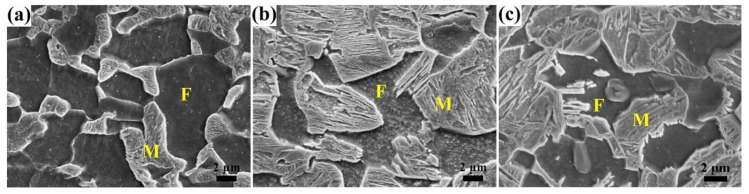

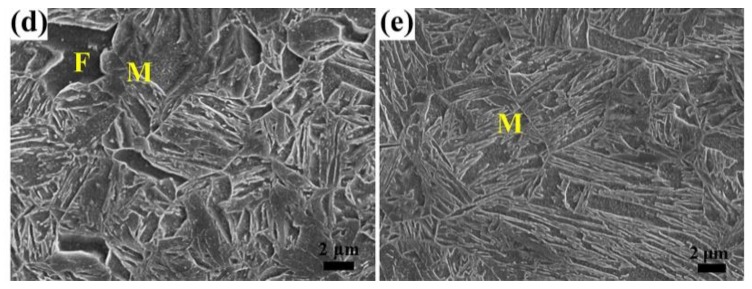
SEM microstructure of high-strength low-alloy (HSLA) steel quenched from (**a**) 790 °C, (**b**) 810 °C, (**c**) 830 °C, (**d**) 850 °C, and (**e**) 900 °C, and tempered at 180 °C. (F = ferrite and M = martensite).

**Figure 2:**
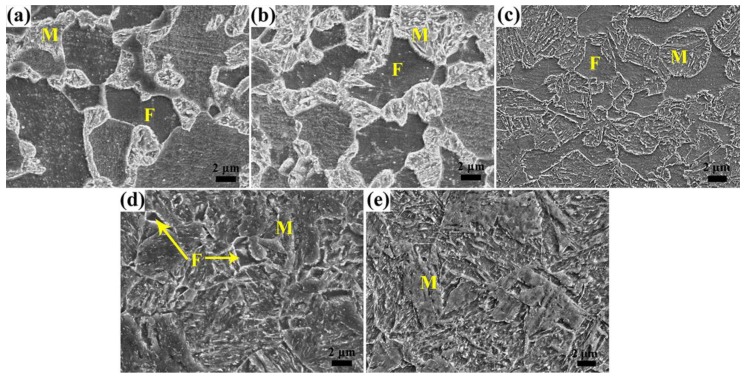
SEM microstructure of HSLA steel quenched from (**a**) 790 °C, (**b**) 810 °C, (**c**) 830 °C, (**d**) 850 °C, and (**e**) 900 °C, and tempered at 450 °C.

The microstructure of the specimens quenched from 790 to 900 °C mainly consists of ferrite and martensite as shown in Figure 1, and the substructures of martensite are clear after tempering at 180 °C. After quenching at 790 °C the ferrite grains are at the large size of ~10 μm and interconnect with each other; martensite islands with size of ~4 μm disperse around the ferrite grains. With increasing quenching temperature, ferrite content decreases from 56 vol% to 0 (see Table 2) with ferrite grain size becoming smaller, while martensite islands become larger and tend to interconnect with each other. Furthermore, when quenching from 850 °C the ferrite content is low at approximately 3 vol% with grain size of ~2 μm and uniformly distributes among the martensite. As the quenching temperature increases to 900 °C, i.e., above A_c3_, the ferrite disappears and the microstructure is complete martensite (see Figure 1e). After tempering at the high temperature of 450 °C, the martensite decomposes dramatically. Its morphology becomes indistinct, with substructures coarsening and a large amount of carbides dispersing in the martensite matrix. The amount of carbides in the martensite increases with increasing quenching temperature (see Figure 2a–e). While, for the ferrite, it changes little with the variation of tempering temperature by comparing the SEM microstructures in Figure 1 and Figure 2.

### 3.2. Tensile Properties

**Figure 3:**
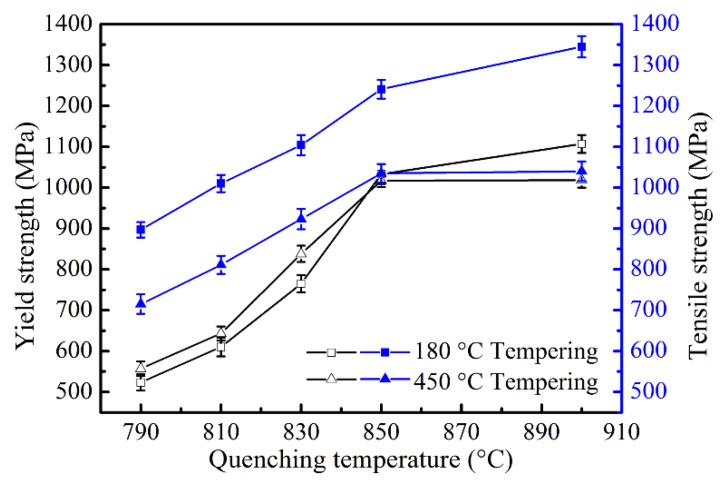
Yield strength and tensile strength as a function of quenching temperature tempered at 180 °C and 450 °C, respectively.

The tensile properties of the specimens after heat treatment were investigated. As shown in Figure 3, both the yield and tensile strengths increase rapidly with increasing quenching temperature (up to 850 °C), where the soft phase ferrite decreases quickly from 56 vol% to 3 vol%, and then rises slightly as the temperature continuously increases to 900 °C, where the ferrite content changes little. This variation is in accordance with the law of mixtures, as shown by Equation (1) [20].

(1) $$\sigma_{t}=\sigma_{m}(1-V_{f})+\sigma_{f}V_{f}$$

where $\sigma_{t}$ is the specimen tensile strength, $\sigma_{f}$ and $\sigma_{m}$ are the ferrite strength and martensite strength, respectively, and $V_{f}$ is the volume fraction of ferrite.

**Figure 4:**
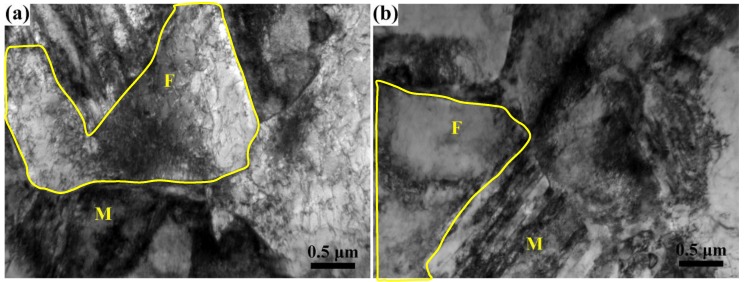
Representative TEM microstructures of specimens with ferrite and martensite quenched from 810 °C and tempered at (**a**) 180 °C and (**b**) 450 °C, respectively.

**Figure 5:**
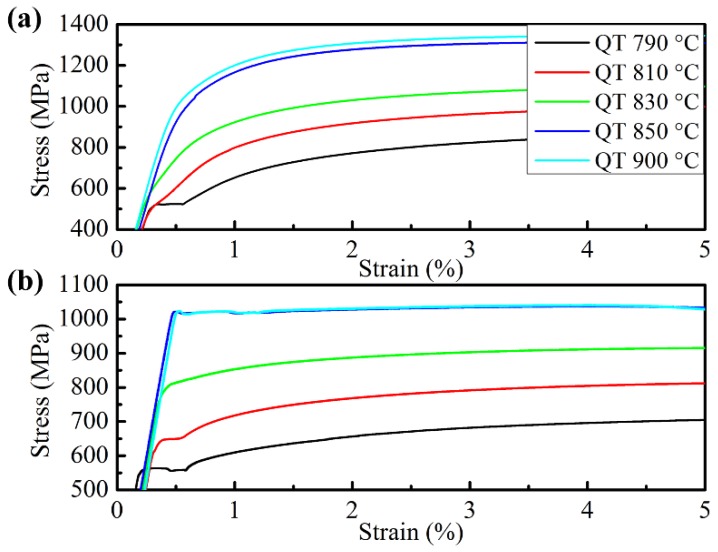
Yielding stage of stress–strain curves of specimens quenched from different temperatures and tempered at (**a**) 180 °C and (**b**) 450 °C, respectively (QT = quenching temperature).

However, the influence of tempering temperature on yield strength and tensile strength is obviously different. The yield strength of the specimens tempered at 450 °C is higher than that tempered at 180 °C when the quenching temperature is below 850 °C, but lower when quenched from 900 °C. This is because when the ferrite fraction is high (>26 vol%), the applied stress is transferred to ferrite grains through the hard martensite and the deformation of ferrite determines the yield strength of the DP steel for its low strength [21]. Meanwhile, the initial martensite transformation introduced many movable dislocations in the adjacent ferrite and its amount is still high even after tempering at 180 °C (see the TEM microstructure in Figure 4a), which makes the ferrite yield under a relatively lower applied stress [22]. The continuous yielding behavior of the specimens in Figure 5a also indicates a large amount of movable dislocations in the microstructures. After tempering at 450 °C, dislocations are pinned by precipitates or slip to tangle with each other as packages locating in the ferrite matrix or near grain boundaries (see Figure 4b), which reduces the amount of movable dislocation resulting in the discontinuous yielding of the stress–strain curves in Figure 5b. So, the onset of plastic deformation of ferrite is successfully suppressed [23,24], and requires a relatively higher applied stress to yield the specimens. Moreover, it is noted that the specimen quenched from 790 °C also shows discontinuous yielding. This is because the initial movable dislocations in ferrite introduced by martensite expansion is low and, after tempering at 180 °C, the dislocations reduce to a lower level which is not enough for the specimen to yield continuously [25]; Marder [26] also pointed out that it needs a minimum amount of martensite transformation to induce enough movable dislocations for the quenched steels to achieve continuous yielding. However, it is still easier for the dislocations to multiply in the 180 °C tempered ferrite than that in the 450 °C tempered ferrite by comparing the yield platforms of the two stress–strain curves quenched from 790 °C. However, the yield strength tempered at 450 °C is lower than that tempered at 180 °C when the ferrite is in low content (3 vol%) and constrained by the interconnected martensite, where the martensite is the main constituent to bear deformation. Even though, in this condition the martensite matrix tempered at 180 °C can yield continuously with enough movable dislocations [27]; it is significantly softened after tempering at 450 °C due to the release of inner stress and matrix decomposition [12,28], so the 450 °C tempered specimens are easier to yield under loading. This is the same condition for the specimens quenched from 900 °C. For the tensile strength, which is determined by the strength of martensite in DP steel, it is lower for the specimens tempered at 450 °C than those tempered at 180 °C in all quenching conditions (see Figure 3). Through the comparison of the specimens quenched from 850 °C and 900 °C, it can be concluded that a small amount of ferrite (3 vol%) almost has no effect on the yield and tensile strength when tempering at high temperature (450 °C), but its influence is significant when the tempering temperature is low (180 °C).

**Figure 6:**
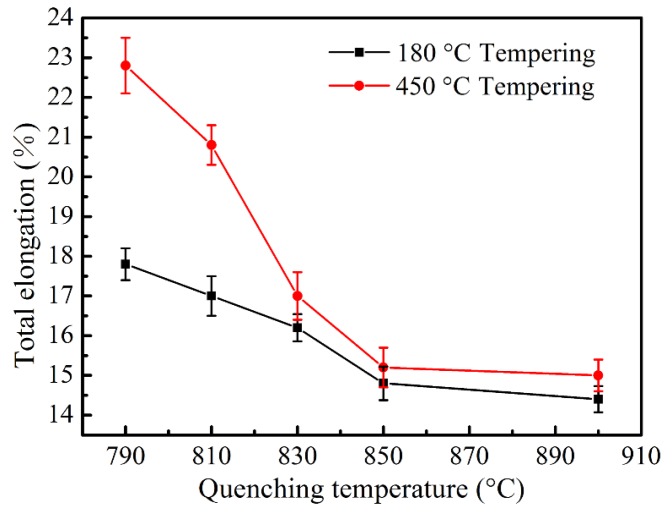
Total elongation as a function of quenching temperature tempered at 180 °C and 450 °C, respectively.

The ductility properties of the specimens after heat treatment are shown in Figure 6. With the quenching temperature increasing to 850 °C, the total elongation decreases rapidly for both tempering conditions due to the fast decrease of ferrite content; the increase of coarse and interconnected martensite around the ferrite (see Figure 1 and Figure 2) promotes the growth of microvoids at a faster rate with less plastic strain by causing severe inhomogeneous deformation [21]. When the quenching temperature is over 850 °C, total elongation changes only slightly due to the similar microstructure for these quenching conditions. The specimens tempered at 450 °C have a better elongation property than those tempered at 180 °C, and the difference becomes significant when the quenching temperature is lower with more ferrite. These probably result from three reasons based on the coordination deformation mechanism of ferrite and martensite. First, with the increase of tempering temperature, the strength of martensite decreases more seriously than ferrite, so the strength difference between them can be reduced which is in favor of the co-deformation of the two constituents [12,29]. Second, the carbon content in martensite, which transfers to austenite from ferrite, decreases at a higher temperature quenching condition, resulting in the reduction of martensite strength [30]. Third, the decrease of ferrite fraction and grain size with the increase of quenching temperature leads to ferrite with higher internal stress introduced by the expansion of martensitic transformation; this also reduces the strength difference between ferrite and martensite and facilitates the coordination deformation between them [6].

**Figure 7:**
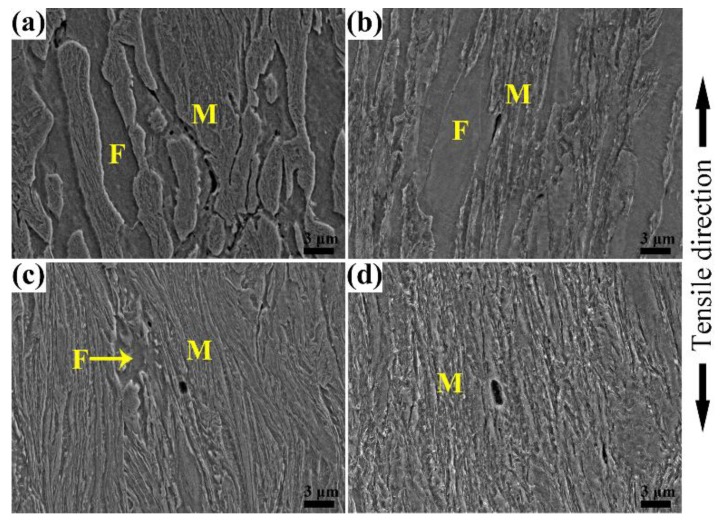
SEM microstructure of deformed HSLA steel next to the tensile fracture surface. (**a**,**b**) with 37 vol% ferrite tempered at 180 °C and 450 °C, respectively, and (**c**,**d**) with 3 vol% ferrite tempered at 180 °C and 450 °C, respectively.

The deformed microstructure next to the tensile fracture surface of specimens after heat treatment was analyzed by SEM as shown in Figure 7. It can be seen that during the tensile process both ferrite and martensite are elongated independent of the ferrite content and tempering temperature. When specimens with a high amount of ferrite (37 vol%) are tempered at 180 °C the strength difference between ferrite and martensite is large, so the ferrite deforms more seriously than martensite, and the constituents’ morphology is easily identified (see Figure 7a). With the increase of tempering temperature the strength difference becomes small, so the martensite deforms coordinately with ferrite; it is hard to identify its morphology due to the serious deformation (see Figure 7b). Microcracks, for both tempering conditions, mainly nucleate at the ferrite–martensite interfaces due to the stress concentration. However, when the specimen contains a low content (<3 vol%) of ferrite and is tempered at 180 °C martensite is the main constituent to bear deformation and the ferrite deforms slightly due to the constraint of martensite as shown in Figure 7c, therefore it can be seen that more ferrite is beneficial for the plastic property. After tempering at 450 °C, martensite has better plasticity and deforms with ferrite more harmoniously, whereas ferrite is elongated with martensite and is difficult to identify (see Figure 7d). Meanwhile, in these two tempering conditions with little ferrite, microcracks mainly nucleate at the martensite packet boundaries for the uniform stress distribution with little ferrite–martensite interface. So it can be concluded that grain boundaries with stress concentration are prior positions for microcracks to nucleate during the tensile process.

### 3.3. Strain Hardening Behavior

**Figure 8:**
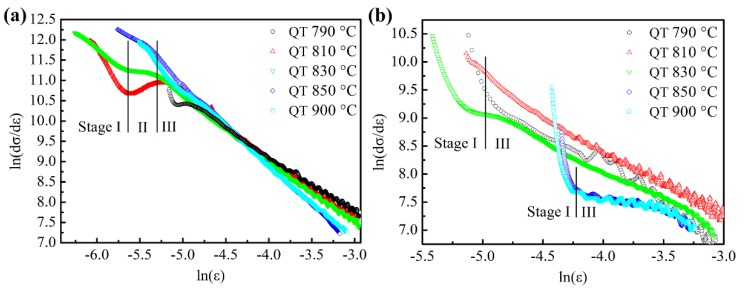
*D*_C–J_ plots of ln(d*σ*/d*ε*) versus ln(*ε*) for specimens quenched from different temperatures and tempered at (**a**) 180 °C and (**b**) 450 °C, respectively.

The strain hardening behavior of the specimens during tensile deformation has been studied by the application of differential Crussard–Jaoul (*D*_C–J_) analysis, which reveals the influence of martensite morphology on strain hardening more effectively at the early stages of deformation for DP steel compared with Hollomon and modified C–J analyses [31]. The *D*_C–J_ analysis is expressed as:

(2) $$\ln\left(\frac{d\sigma }{d\varepsilon }\right)=\left(n-1\right)\ln\varepsilon +\ln(kn)\;$$

which is based on the power Ludwik relation:

(3) $$\sigma =\sigma_{0}+{k\varepsilon }^{n}$$

where σ is the true stress, ε is the true strain, *n* is the strain hardening exponent, and $\sigma_{0}$ and *k* are material constants. The results of the *D*_C–J_ analysis of tensile dates are shown in Figure 8. The values of *n* − 1 at different stages calculated by fitting the slopes of curves for all of the specimens are summarized in Table 3. After tempering at 180 °C, the results of the strain hardening for specimens quenched from 790 °C, 810 °C, and 830 °C show three stages (labeled as stage I, II, and III) according to the values of *n* − 1 (see Figure 8a), which reveals different deformation mechanisms [25,31,32]. The first stage I is attributed to the homogeneous deformation of the soft ferrite matrix induced by the glide of mobile dislocations or residual stresses surrounding the martensite regions. The second stage II is attributed to the deformation of the constrained ferrite and possible transformation of retained austenite into martensite. Stage III is attributed to the simultaneous deformation of ferrite and martensite. When quenching from 850 °C, the plots of strain hardening show two stages (II and III), which indicates that the ferrite in the microstructure is constrained by martensite and is hard to deform (see Figure 7c). When the quenching temperature increases to 900 °C, only stage III is evident, as expected, due to the deformation of single-phase martensite. These changes are similar to those results of DP steels without tempering in the literature [25,31]. In Table 3, the values of *n* − 1 in stage I increase with the quenching temperature increasing from 790 °C to 830 °C due to the increasing movable dislocation density in the ferrite matrix along the ferrite–martensite interfaces introduced by the expansion of martensite transformation. However, in the third stage, the values of *n* − 1 decrease with the increase of martensite volume fraction and this result agrees with other researches [22,32].

However, the specimens tempered at 450 °C with different ferrite content show two-stage (I and III) strain hardening behavior as shown in Figure 8b, which indicates that all specimens have the same strain hardening behavior after tempering at high temperature, i.e., the ferrite and martensite deform coordinately in both stages I and III due to the decrease in inner stress and the strength difference between them. The values of *n* − 1 in stage I tempered at 450 °C are lower than that tempered at 180 °C (see Table 3), which indicates lower movable dislocation density in the specimens at the beginning of strain hardening, because at the discontinuous yielding stage the pinned dislocations are slip-resistant. The occurrence of discontinuous yielding retards the strain hardening process, e.g., the beginning of plots quenched from 790 °C is later than the others as shown in Figure 8a, so it is clear that the strain hardening process begins only when adequate movable dislocations appear during deformation. Otherwise, the values *n* − 1 in stage I tempered at 450 °C decrease with increase of quenching temperature. This may be attributed to that as the amount of cementite precipitating in martensite increases with the quenching temperature increasing from 790 °C to 900 °C (Figure 2), the dislocations in the vicinity of precipitates during deformation are accelerated to multiply and then interact with each other and annihilate, so the strain hardening rate is either enhanced by dislocation multiplication or reduced by dynamic recovery [33]. With the deformation going on, dislocations tangle with each other and form dislocation cells and walls, i.e., statistically stored dislocation, and the movement of the statistically stored dislocation determines the work hardening rate. Thus the higher volume fraction of martensite, with more precipitates hindering the statistically stored dislocations to move, enhances the work hardening rate [34], which is consistent with the value variation of *n* − 1 in stage III tempered at 450 °C, as shown in Table 3. Finally, it can be concluded that the amount of mobile dislocation and precipitates together determines the strain hardening behaviors of the HSLA steel.

### 3.4. Impact Properties

**Figure 9:**
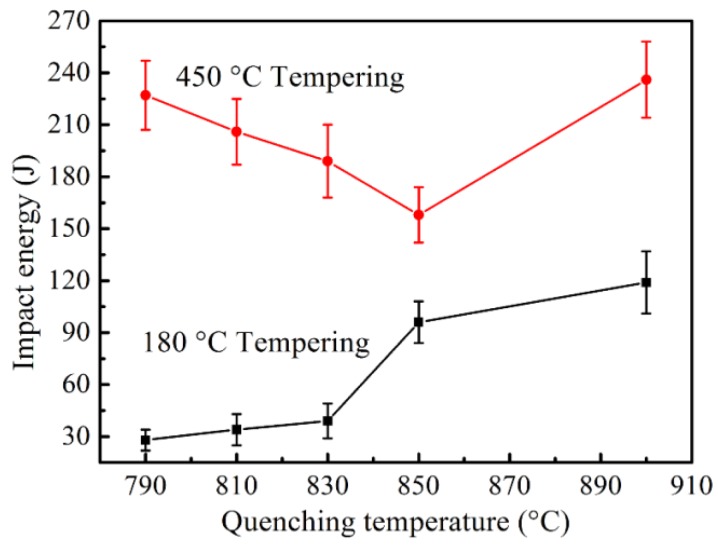
−40 °C Charpy V-notched impact energy versus quenching temperature of the specimens tempered at 180 °C and 450 °C, respectively.

**Figure 10:**
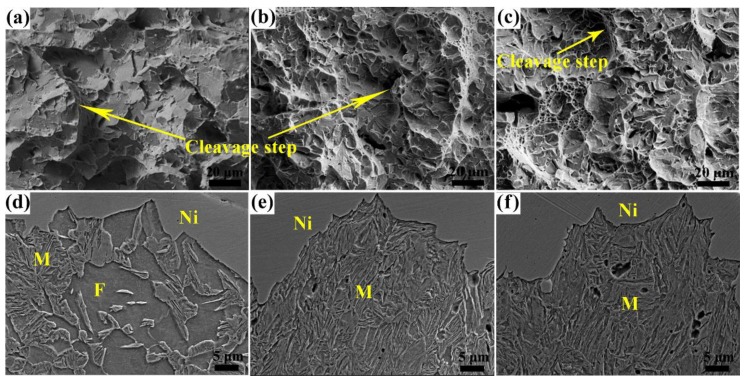
SEM images of the −40 °C impact fracture morphology and microstructure underneath the fracture surface of the specimens tempered at 180 °C with ferrite content of (**a**,**d**) 37 vol%, (**b**,**e**) 3 vol%, and (**c**,**f**) none, respectively. (Ni = coated nickel layer).

The values of −40 °C average Charpy V-notched impact energy (*Ec*) of the specimens tempered at different temperatures are plotted against the quenching temperature in Figure 9. After tempering at 180 °C, the *Ec* increases with the increase of quenching temperature and the specimens with ferrite <3 vol% have much better toughness (*Ec* > 96 J) than those with ferrite >26 vol%, which agrees with the results of Yang [35]. While this change of toughness is in contrast with the variation trend of the elongation for the specimens tempered at 180 °C (see Figure 6), it is generally known that the soft ferrite can improve the ductility and toughness [2,36]. This may be attributed to the different fracture mechanisms of the tensile process and impact after tempering at low temperature, as shown in Figure 10. During the impact process, both ferrite and martensite near the fracture surface almost do not deform, and microcracks mainly nucleate in the ferrite matrix and at the martensite packet boundaries and then expand through the ferrite grains, while the crack expansion path changes at the martensite grain boundaries (see Figure 10d), which indicates that the ferrite provides a crack propagation channel while hard martensite hinders crack growth. The cleavage surface and steps in Figure 10a also prove this impact fracture mechanism. With the decrease of ferrite content, the crack extended path in ferrite reduces or even disappears (see Figure 10e,f), so the cracks become more tortuous due to the hindrance of martensite, and this can also be proved by the smaller cleavage facets and dimples in Figure 10b,c, which indicate a higher amount of energy consumption by a greater proportion martensite for microcrack nucleation and growth before fracture [37].

**Figure 11:**
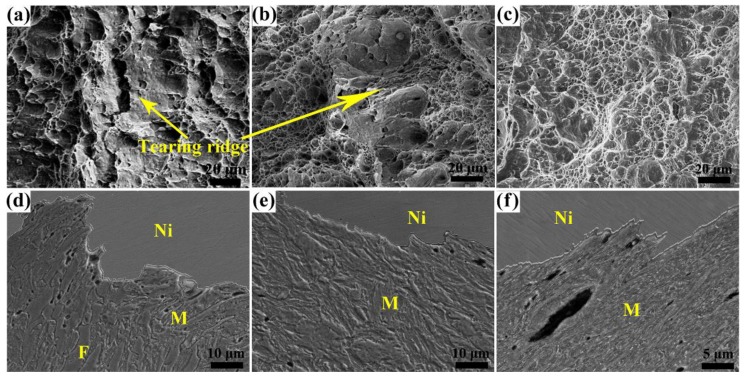
SEM images of the −40 °C impact fracture morphology and microstructure underneath the fracture surface of the specimens tempered at 450 °C with ferrite content of (**a**,**d**) 37 vol%, (**b**,**e**) 3 vol%, and (**c**,**f**) none, respectively.

After tempering at 450 °C, the values of *Ec* are much higher than those tempered at 180 °C and decrease firstly with increasing quenching temperature, with the bottom at ~850 °C with little ferrite, and then an increase at 900 °C (see Figure 9). This variation, which differs from the one tempered at 180 °C, keeps in line with the trend in the elongation of the specimens tempered at 450 °C. This is because of that the impact fracture mechanism is similar to the tensile fracture mechanism, that both ferrite and martensite have good plasticity and are elongated during impact process for their coordination deformation as shown in Figure 11d–f. When the ferrite content is high (37 vol%), microcracks mainly nucleate at ferrite–martensite interfaces due to the stress concentration and then join up to extend forward along these interfaces, and the martensite and ferrite next to the fracture have large plastic deformation, which indicates that more impact energy is required to break the specimen; the tearing ridges on the fracture surface in Figure 11a also reveal the fracture mechanism [24]. So the reduction of ferrite content decreases the microstructural deformability resulting in low impact energy. However, when the ferrite content is reduced to zero (quenching from 900 °C) the impact energy is much higher than that with 3 vol% ferrite. This can be explained by that when there is only tempered martensite, all the microstructures can equally bear the impact stress, so the microcracks evenly nucleate at martensite packet boundaries (see Figure 11f) and without stress concentration at one place it needs more energy to drive the cracks to extend through the martensite. The dimples on the fracture surfaces of specimens without ferrite are more uniform than those with little ferrite (see Figure 11b,c) which also indicates the uniform crack extension in the former.

## 4. Conclusions

The synergetic effects of ferrite content and tempering temperature on tensile and impact behaviors of a Q960E HSLA steel have been investigated. The main conclusions are summarized as follows.

1. The heat treated microstructure mainly consists of ferrite and martensite and the ferrite content varies from 56 vol% to 0 with quenching temperature increasing to 900 °C; the martensite decomposes seriously with less dislocations and more carbides when tempering at 450 °C, which leads to the appearance of a yielding point;
2. High ferrite content reduces the yield strength and tensile strength based on the law of mixtures, and tensile strength decreases with the increase of tempering temperature, while the yield strength increases for specimens with >26 vol% ferrite and decreases for specimens with <3 vol% ferrite;
3. When tempering at 180 °C, specimens show different strain hardening behaviors with the variation of ferrite content, and ferrite improves the elongation but deteriorates the toughness with different fracture mechanisms due to the strength difference between ferrite and martensite. However, all specimens show same strain hardening behavior due to the co-deformation of ferrite and martensite after tempering at 450 °C, and the addition of ferrite benefits both elongation and impact properties with similar fracture mechanisms. Specimens quenched from 900 °C with complete martensite have better toughness over 119 J after tempering at both temperatures.

## Figures and Tables

**Table 1:** Chemical composition of the investigated steel (wt %).

Chemical Composition	Contents (wt %)
**C**	0.12
**Si**	0.30
**Mn**	1.30
**Cr**	0.25
**Mo**	0.10
**Nb**	0.02
**Ti**	0.017
**P**	0.015
**S**	0.001
**Fe**	Bal.

**Table 2:** The volume fraction of ferrite quenched from different temperatures.

Quenching Temperature/°C	Ferrite Content/vol%
790	56
810	37
830	26
850	3
900	0

**Table 3:** Summary of the parameters related to strain hardening behavior of each heat treated specimens.

Quenching Temperature/°C	Tempering Temperature/°C	Slope (*n* − 1)		
		Stage I	Stage II	Stage III
790	180	−5.55	−0.42	−1.50
810		−3.38	0.95	−1.64
830		−1.69	−0.21	−1.62
850		−−	−1.15	−1.99
900		−−	−−	−1.94
790	450	−5.59	−−	−1.09
810		−4.20	−−	−1.34
830		−4.70	−−	−1.03
850		−9.29	−−	−0.48
900		−12.52	−−	−0.39
